# Iatrogenic Pneumothorax during Acupuncture: Case Report

**DOI:** 10.3390/medicina59061100

**Published:** 2023-06-07

**Authors:** Wen-Shan Chiu, Yu-Wen Lu, Ting-Hsuan Lien

**Affiliations:** Department of Chinese Medicine, Show Chawn Memorial Hospital, Changhua 500, Taiwan

**Keywords:** acupuncture, iatrogenic pneumothorax, chronic pulmonary diseases, imaging

## Abstract

Acupuncture treatment in local areas is commonly used to treat pain or soreness; however, acupuncture around the neck or shoulder may be a risk factor for pneumothorax. Herein, we report two cases of iatrogenic pneumothorax after acupuncture. These points indicate that physicians should be aware of these risk factors through history-taking before acupuncture. Chronic pulmonary diseases, such as chronic bronchitis, emphysema, tuberculosis, lung cancer, pneumonia, and thoracic surgery, may be associated with a higher risk of iatrogenic pneumothorax after acupuncture. Even if the incidence of pneumothorax should be low under caution and fully evaluated, it is still recommended to arrange further imaging examinations to rule out the possibility of iatrogenic pneumothorax.

## 1. Introduction

Poor lifestyle habits and posture are common causes of shoulder and neck pain. As modern people work long hours and frequently use electronic devices, such as computers and mobile phones, chronic shoulder and neck pain has become a civilized illness. Acupuncture and moxibustion significantly alleviate chronic shoulder and neck pain [1,2]. Some physicians use electro-acupuncture to enhance this effect. Acupuncture, an ancient healing practice originating in China, has become widely used as a complementary and alternative medicine globally [3,4,5]. This technique involves the insertion of fine needles made of gold, silver, or stainless steel into specific acupoints on the body to alleviate symptoms [3,4]. Acupuncture points commonly used to treat shoulder and neck pain include Jianjing (GB21), Fengchi (GB20), Wangu (GB12), Jianzhongshu (SI15), Bingfeng (ST12), Quchi (LI17), Tianzong (SI11), Jianwaishu (SI14), Tianzhu (BL10), Dazhu (BL11), Fengfu (GV16), DaiZhui (GV14), Futu (LI18), and Tianchuang (SI16). Most of these points are the gallbladder, bladder, and small intestine meridians located in the shoulder and neck areas [6,7].

Acupuncture in local areas is a common method to treat pain or soreness; however, that around the neck or shoulder area may be a risk factor for pneumothorax [8,9]. Pneumothorax is defined as the presence of air or gas in the pleural space, which is the space between the lungs and chest wall. Pneumothorax is classified, based on its etiology, primarily into two categories: spontaneous and acquired, which are further subcategorized as traumatic or iatrogenic [10]. According to the previous statistical data, the proportion of pneumothorax caused by acupuncture in “at-risk” anatomical areas is twice as high as that of acupuncture in safe areas [11]. This result indicates that performing acupuncture on the shoulder is dangerous. However, there is still the possibility of spontaneous pneumothorax while performing acupuncture in other areas. Serious and potentially life-threatening adverse events associated with acupuncture are rarely reported. These include pneumothorax, cardiovascular damage, bleeding, or bruising of the central nervous system [9]. Prospective studies have reported approximately 0.001% incidence of pneumothorax following acupuncture [12,13]. In a large study conducted in Taiwan, the rate was 0.87 per million sessions; even at high-risk sites, it was 1.75 per million sessions, indicating that the occurrence of pneumothorax is extremely rare [11]. However, pneumothorax is relatively common among serious adverse events such as organ damage and death. Here, we report two cases of iatrogenic pneumothorax after acupuncture. We will focus on imaging studies and discussions.

## 2. Case Presentation

### 2.1. Case 1

A 21-year-old female college student visited the Chinese Medicine Outpatient Clinic of our hospital because of an abnormal menstrual cycle. Her height and weight were 160 cm and 60 kg, respectively (Body Mass Index (BMI): 23.43 kg/m^2^), and she denied any past systemic disease. During recuperation, the patient received acupuncture for shoulder and neck pain treatment. The bilateral acupoints GB21 (Jianjing) and GB20 (Fengchi) were used. A few minutes after the removal of the needle, the patient experienced slight chest tightness and discomfort. Needle shock, also known as vasovagal syncope, is a common adverse event of acupuncture treatment, was suspected. Owing to the shallow depth of the acupoint GB21 (Jianjing), pneumothorax was ruled out. After the chest tightness subsided, she returned home.

**Figure 1:**
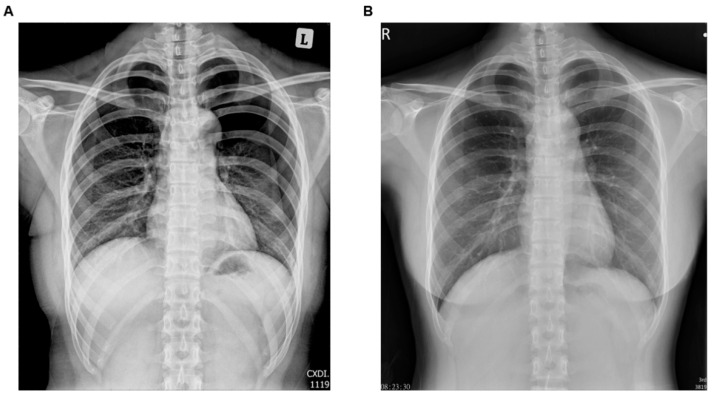
Chest X-ray (CXR) (**A**) after acupuncture treatment, the next day, and (**B**) when the patient was discharged from the hospital.

The next day, the patient complained that the left-sided chest tightness was exaggerated while performing deep breathing. The patient then visited our hospital for further evaluation. Her vital signs were as follows: body temperature, 36.5 °C; blood pressure, 108/85 mmHg; pulse rate, 85 beats/min; and breathing, 20 beats/min. She had no specific abnormality under inspection; however, chest fremitus occurred and bilateral lung breath sounds weakened. Left chest percussion revealed hyper-resonance compared to the right side. A chest X-ray (CXR) revealed bilateral upper lung pneumothorax and approximately 50% lung tissue collapse (Figure 1A). A bilateral pneumothorax was suspected, and thoracic surgery was performed. During hospitalization, left thoracic catheter drainage and nasal inhalation of pure oxygen were performed. After 5 days, CXR showed improvement in the pneumothorax (Figure 1B). The patient was discharged without chest discomfort and with stable vital signs.

### 2.2. Case 2

**Figure 2:**
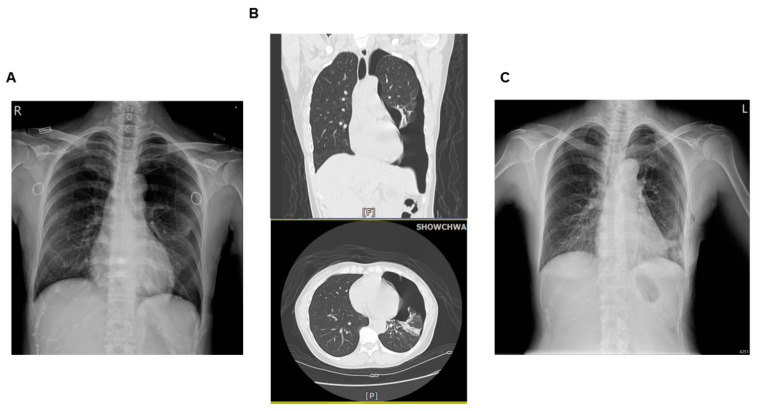
(**A**) Chest X-ray (CXR) and (**B**) computed tomography (CT) after acupuncture treatment (F: Front; P: Posterior). (**C**) CXR taken when the patient was discharged from the hospital.

A 53-year-old woman with a height and weight of 163 cm and 49 kg (BMI: 18.4 kg/m^2^), respectively, underwent long-term traditional Chinese medicine (TCM) therapy because of pain and stiffness of the right shoulder and trapezius muscles. On 4 May 2021, the TCM physician applied acupuncture and electroacupuncture to the patient’s shoulders, back, and GB21 (Jianjing) points on both sides. The patient complained of chest tightness, pain, and shortness of breath during needle retention. The nurse immediately turned off the electroacupuncture machine and extracted the needles. A pneumothorax was suspected, and a CXR was obtained for further evaluation. Her vital signs showed a body temperature of 35.8 °C, blood pressure of 183/97 mmHg, pulse of 59 beats/min, and respiration of 20 times/min. The CXR revealed a left lower lobe pneumothorax (Figure 2A). Chest computed tomography (CT) and video-assisted thoracoscopic surgery (VATS) with removal of bullae (bullectomy) were arranged the next day, as the CT scans showed lung tissue with subpleural emphysematous bullae under the visceral pleura (Figure 2B). Five days later, the CXR showed improvement in the pneumothorax, and vital signs were stable (Figure 2C). There was no chest tightness or discomfort, and the patient was discharged.

## 3. Discussion

In these two cases, the thickness of the patient’s muscle layer was determined using CT imaging. The distances from the thoracic cavity to the chest cavity in cases 1 and 2 were approximately 4.5 and 4 cm, respectively. The acupoint GB21 is situated at the pinnacle of the trapezius muscle in the sagittal plane, and corresponds to the highest point of the lung, which bilaterally forms a dome. The accuracy of needle insertion is crucial as the lung surface was reported to lie between 10.1 to 29 mm in men, and 12 to 20 mm in women beneath the skin [14]. Due to relying on the recommendations of traditional acupuncture books for needle insertion depth, female patients may experience greater susceptibility to pneumothorax following GB21 acupuncture than male patients [14]. The acupuncture needles commonly used in GB21 (Jianjing) points were 1 (2.5 cm) and 1.5 inches (4 cm), and the diameter is between 0.2 to 0.27 mm [8]. According to current research reporting on the safe depth using the GB21 (Jianjing) acupoint, the average depth of the GB21 acupoint is approximately 3.7–7 cm according to different body weights [15,16]. However, in clinical practice, judging the depth of acupuncture based on body weight is challenging, and a misdiagnosis may occur. Furthermore, a safe depth is related to posture. For acupuncture at GB21, the prone position is safer than sitting [17].

Owing to the elasticity of the alveoli of muscle tissue, most iatrogenic pneumothorax caused by acupuncture is pneumothorax, in which the area of the lung shrinks by less than 20% [11]. The rest is required in most cases, and no specific therapeutic intervention is required. Therefore, Chinese medicine practitioners should consider these two cases as warnings. Although there was no clear evidence from the imaging studies, the acupuncture needles in these two cases could not break through the muscle layer. However, because the mechanism of action of acupuncture is unknown, there is a considerable risk of iatrogenic pneumothorax.

Healthcare professionals should maintain a high level of suspicion of hemothorax, pneumothorax, or both when a patient reports dyspnea, chest pain, or interscapular pain following acupuncture in the thoracic area [18]. Pneumothorax may require invasive intervention and ongoing monitoring because it has the potential to develop into a critical condition that may compromise cardiovascular function. Prompt medical recognition and intervention are crucial to achieving the best possible patient outcomes [19]. Spontaneous pneumothorax is commonly observed in underweight young males and patients with lung abnormalities. Risk factors include chronic obstructive pulmonary disease, cystic fibrosis, malignancy, infections, cystic lung disorders, catamenia (endometriosis), architectural abnormalities (Marfan syndrome), anorexia, exercise, and illicit or immunosuppressant drug use [20]. Associated with the conditions of the lungs and thorax, patients with chronic pulmonary diseases, such as chronic bronchitis, emphysema, tuberculosis, lung cancer, pneumonia, and thoracic surgery may have a higher incidence of pneumothorax after acupuncture [11]. Subpleural emphysematous bullae were found in case 2 after thoracoscopic surgery. Although the patient had not been previously diagnosed with lung disease, an abnormal lung is often an important risk factor for pneumothorax. For patients who are at risk, such as those with a history of acupuncture treatment, it may be advisable to include this information in the medical history assessment of those who present with symptoms such as chest pain and/or breathing difficulty.

Thoracic endometriosis is a neglected risk factor due to its extremely low probability [21]. An ancient Chinese medical reference described atopomenorrhea, which means coughing up or vomiting blood during menstruation, similar to intrathoracic endometriosis in modern medicine. Case 1 experienced menstrual pain and underwent acupuncture during menstruation. However, judging from the imaging of the bilateral collapse of her lungs, she possibly had medical pneumothorax caused by acupuncture. In most cases (95%), the right hemithorax is involved, while the left hemithorax is involved in only 5% of the cases, and bilateral involvement is quite rare [22]. However, attention should be paid to the history of menstruation because endometriosis is a risk factor for spontaneous pneumothorax [23].

Considering the risk of acupuncture in the shoulder area, we considered alternative methods to replace needling. If the same invasive treatment is used, dry needling is considered to stimulate the trigger points of the shoulder myofascial [24]. Cupping is also a good alternative if patients select a more conservative treatment method [25]. Cupping causes local hyperemia, promotes blood circulation, regenerates local tissue, and relieves pain. Cupping has become popular since many international sporting events have introduced it to treat athletes. Additionally, some physicians choose distal acupoints to treat the shoulder, such as the ear and scalp [26]. According to bioholography theory, many parts of the body have corresponding regions. Other manual treatments and physical therapies can also help relieve shoulder pain. Therefore, needles should be avoided in high-risk areas that are not completely secure.

## 4. Conclusions

We reported two cases of iatrogenic pneumothorax that occurred with acupuncture which indicated that physicians should be aware of the risk factors through history-taking before acupuncture. From the imaging of two patients, it was concluded that the acupuncture needle length did not exceed the thickness of the chest wall. However, judging from the timeline, acupuncture was the trigger of the pneumothorax. Chronic pulmonary diseases such as chronic bronchitis, emphysema, tuberculosis, lung cancer, pneumonia, and thoracic surgery may be associated with a higher risk of iatrogenic pneumothorax after acupuncture [11]. Patients with such medical histories should avoid chest and back acupuncture. Acupuncture applied to parts other than the chest and back should also be arranged in a prominent position so that physicians can detect any discomfort in patients at any time.

Even if the incidence of pneumothorax should be low under caution and fully evaluated, it is still recommended to arrange further imaging examinations to rule out the possibility of iatrogenic pneumothorax. Although many studies have been conducted regarding the depth of acupuncture points, clinicians are still unable to apply the average acupoint depth to each patient because of large individual differences. Therefore, vertical needle insertion into the shoulder or back should be avoided. Instead, cupping, hypodermic needles, distal acupuncture, or other methods should be used. If necessary, imaging should be considered to assist in diagnosing the depth of acupuncture and avoid the risk of iatrogenic pneumothorax.

## Data Availability

All data and material are presented in the manuscript.
